# Supplementary material for: The impact of obesity-related raised intracranial pressure in rodents
Source: Sci Rep. 2022 Jun 1;12:9102. doi: 10.1038/s41598-022-13181-6 (PMC9160066; doi:10.1038/s41598-022-13181-6)
Supplement: Supplementary file 1 — Supplementary Information. [file 41598_2022_13181_MOESM1_ESM.docx]

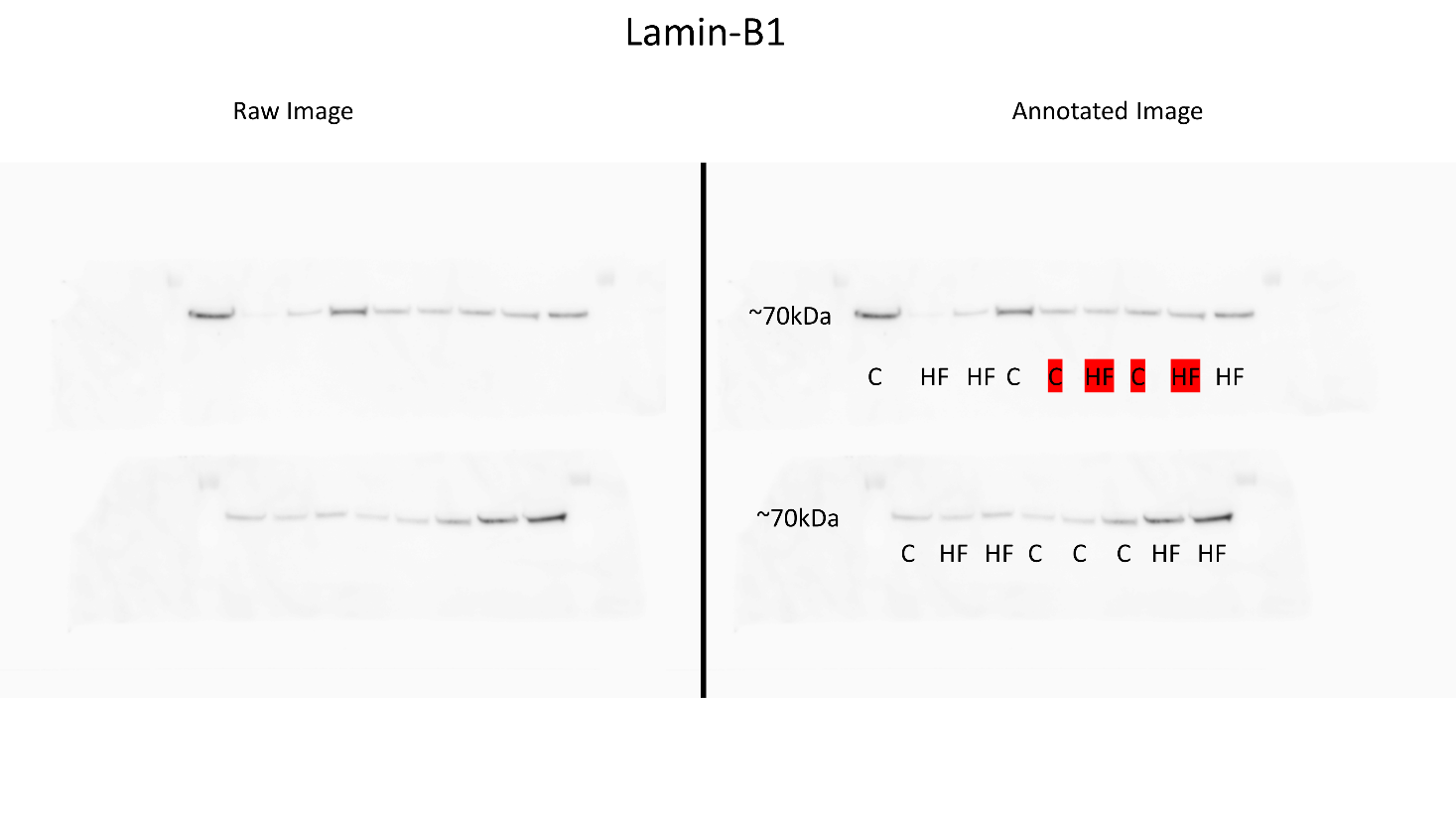
Supplemental 1. Raw Lamin B1 immunoblots

Lamin-B1 immunoblots ran in parallel, where membranes were blotted in parallel and were cut according to molecular weight prior to immunoblotting. For the manuscript the membranes were cropped to show representative bands. Red highlighted letters show the columns presented in the manuscript. Annotated image shows group allocation of each well, C= control, HF= high fat. The image also contains the molecular weights.


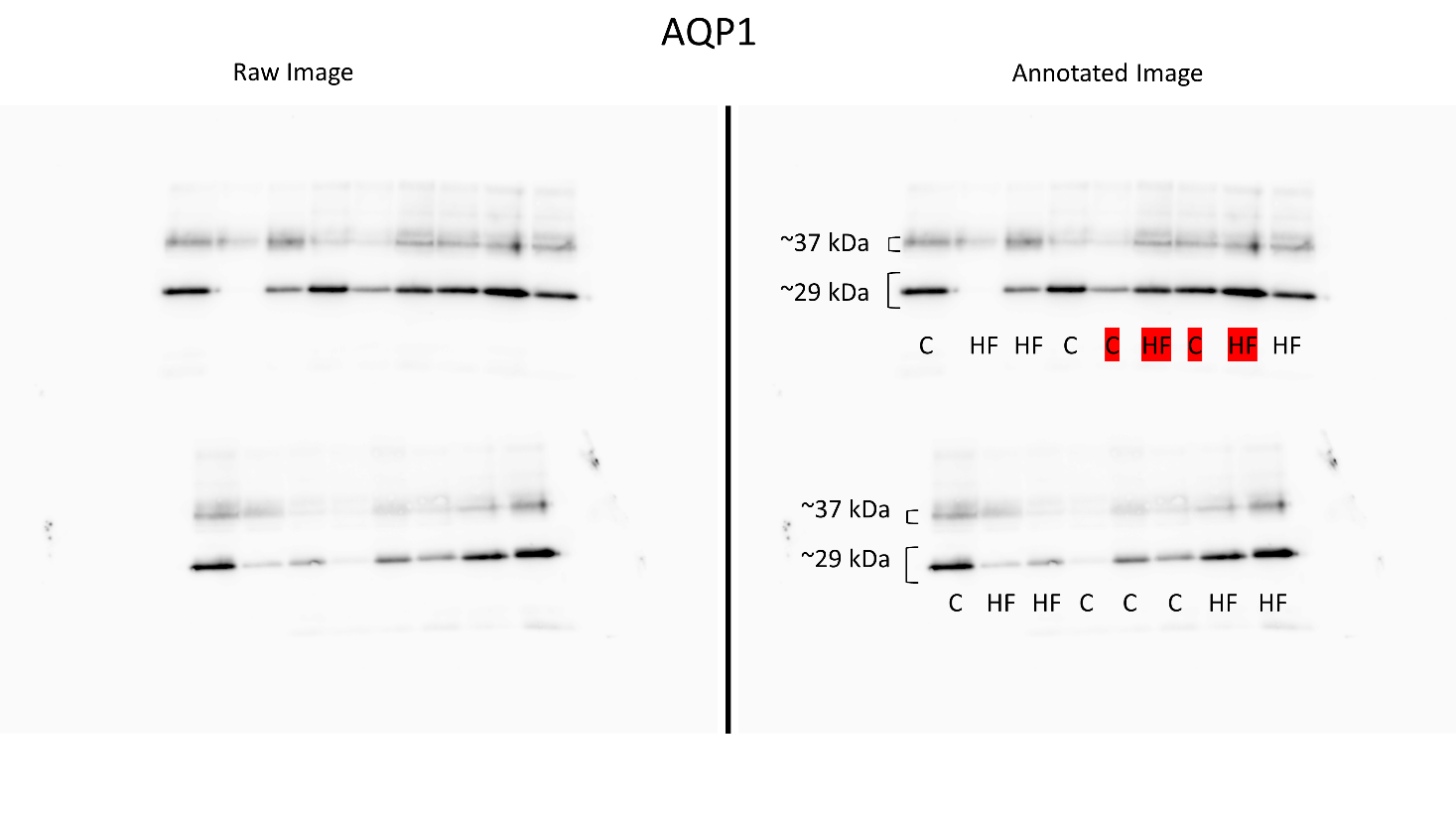


Supplemental 2. Raw AQP1 immunoblots

AQP1 immunoblots ran in parallel, where membranes were blotted in parallel and were cut according to molecular weight prior to immunoblotting. For the manuscript the membranes were cropped to show representative bands. Red highlighted letters show the columns presented in the manuscript. Annotated image shows group allocation of each well, C= control, HF= high fat. The image also contains the molecular weights.


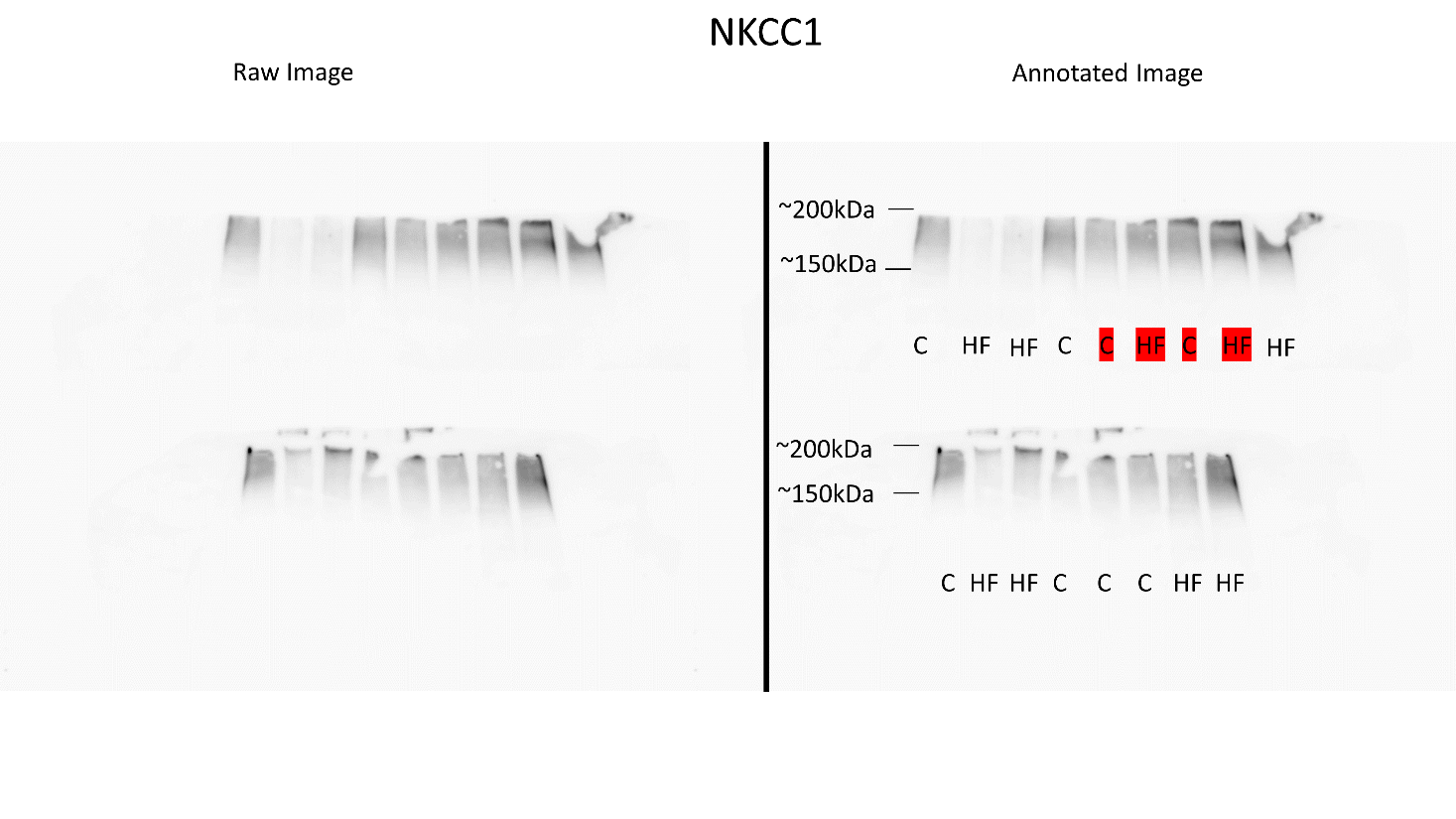


Supplemental 3. Raw NKCC1 immunoblots

NKCC1 immunoblots ran in parallel, where membranes were blotted in parallel and were cut according to molecular weight prior to immunoblotting. For the manuscript the membranes were cropped to show representative bands. Red highlighted letters show the columns presented in the manuscript. Annotated image shows group allocation of each well, C= control, HF= high fat. The image also contains the molecular weights.
